# Supplementary material for: MRI-guided robotic arm drives optogenetic fMRI with concurrent Ca2+ recording
Source: Nat Commun. 2019 Jun 10;10:2536. doi: 10.1038/s41467-019-10450-3 (PMC6557837; doi:10.1038/s41467-019-10450-3)
Supplement: Supplementary file 1 — Supplementary Information [file 41467_2019_10450_MOESM1_ESM.pdf]

Supplementary Information

MRI-guided robotic arm drives optogenetic fMRI with concurrent Ca^{2+} recording

Chen et al.

Supplementary Table (1)

Supplementary Table 1. List of Components of MgRA

Name	Information
Archimedean spiral driving design	Custom-designed, MPI for BC Mechanic Workshop
Bearings for back/forward	JSM-2022-20, Igus, Germany
Bearings for left/right	BB-625-B180-10-GL, Igus, Germany
Belts	Optibelt OMEGA 3M, OPTIBELT, Germany
Camera	RS-OV7949-1818, Conrad Electronic, Germany
Carbon fiber tube (1)	7420182, R&G, Germany
Carbon fiber tube (2)	7420173, R&G, Germany
Charging condenser	Z-K4700/50, Nanotec, Germany
Cross table	Custom-designed, MPI for BC Mechanic Workshop
Encoder cable	ZK-NOE1-10-20000-S, Nanotec, Germany
Encoders	NOE2-05-B14, Nanotec, Germany
Gearbox	GPLE22-2S-12, Nanotec, Germany
Matching toothed pulley	Custom-designed, MPI for BC Mechanic Workshop
Motor controller	SMCI33-1, Nanotec, Germany
Platform for MgRA	Custom-designed, MPI for BC Mechanic Workshop
Power supply	NTS-24V-40A, Nanotec, Germany
Rat holder	Custom-designed, MPI for BC Mechanic Workshop
Robotic arm holder	Custom-designed, MPI for BC Mechanic Workshop
Stepper motor	ST4118D1804-B, Nanotec, Germany
USB cable for motor controller	ZK-USB, Nanotec, Germany
Cable lengthening	Custom-designed, MPI for BC Electronic Workshop
Other pieces of MgRA	Custom-designed, MPI for BC Mechanic Workshop

Supplementary Figures (13)

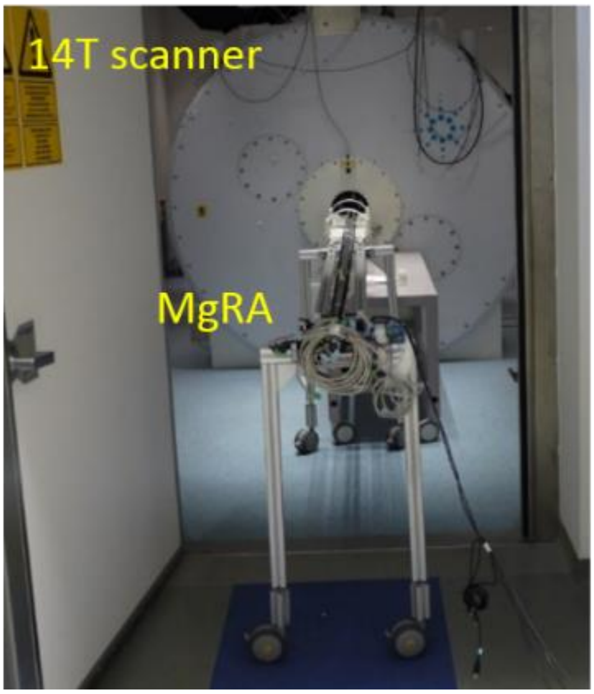

Supplementary Figure 1. MRI compatible MgRA mounted inside the 14T MRI scanner (picture taken from the control room).

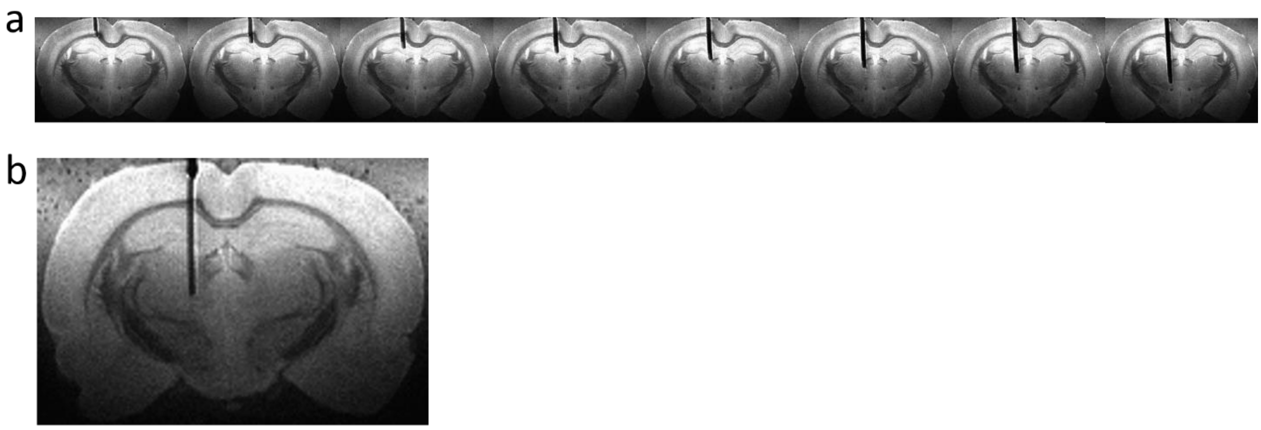

Supplementary Figure 2. Examples of optical fibers with different core diameters driven by MgRA for *in vitro* evaluation. **a** The T2-weighted MRI images show 8 different locations of the optical fiber (400 μm core diameter, black stripe) along the insertion trajectory in a perfused rat brain embedded in the soft agarose with manganese. **b** Optical fiber (200 μm core diameter, black stripe) was inserted vertically into the perfused rat brain.

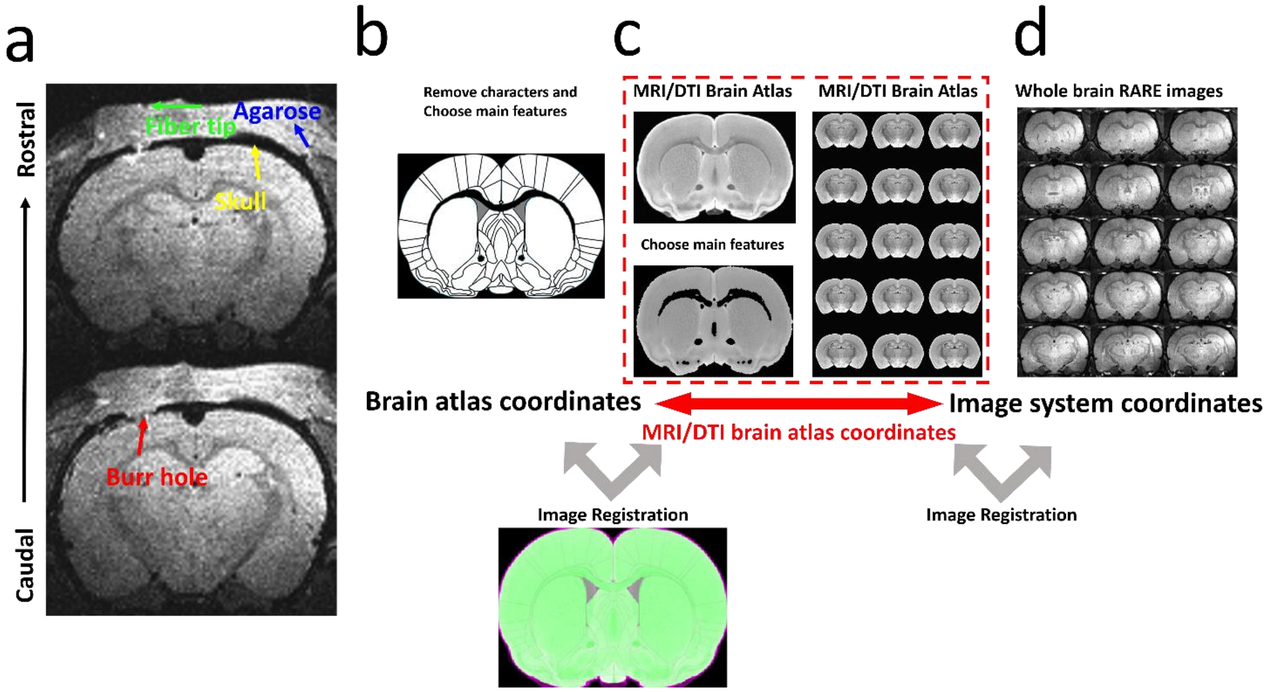

Supplementary Figure 3. MRI-based relocation outside of the rat brain and the registration of coordinates. **a** Agarose with manganese was applied to cover the skull (yellow arrow). By lowering the fiber into the agarose, we could calculate the distance between fiber tip (green arrow) and burr hole (red arrow) from the anatomical images. The burr hole is filled with agarose as well. **b** Atlas coordinates (Co1). **c** MRI/DTI Atlas of the Rat Brain (Co2, provided by Dr. G. Allen Johnson). **d** 3D anatomical images of an individual rat.

For coordinates registration, agarose has been previously applied above the burr hole of the skull and the fiber tip (previously positioned above the burr hole using the MgRA system under the guidance of the build-in camera inside the MR scanner) can be directly imaged to determine its coordinates in the MRI images (Supplementary Figure 3a). Then, an algorithm was designed to register a four coordinate system for the fiber tip position: atlas coordinate (Co1), MRI/DTI rat brain atlas (Co2, provided by Dr. G. Allen Johnson), MRI coordinate (Co3) and robotic arm coordinate (Co4). In short, the Co1 is first transferred to the Co2 by the algorithm (Supplementary Figure 3b, c). By registering the 2D anatomical images of individual rat (Co3) to the MRI/DTI brain atlas (Co2), the transformation between the Co1 and Co3 is settled (Red arrow in Supplementary Figure 3c). Since the fiber tip position is directly detected in the MRI images above the craniotomy, the related coordinate offset from the fiber tip to the targeted function nuclei can be calculated based on the multiple transformation matrices.

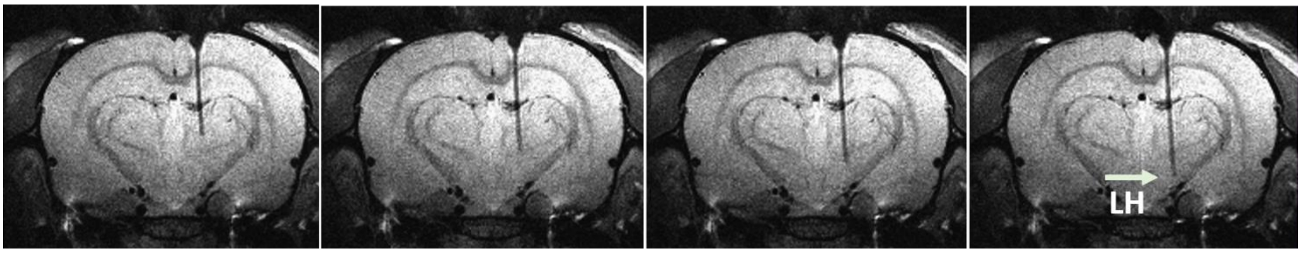

Supplementary Figure 4. The time-lapsed anatomical images to illustrate the optical fiber targeting the Lateral Hypothalamus for opto-fMRI studies.

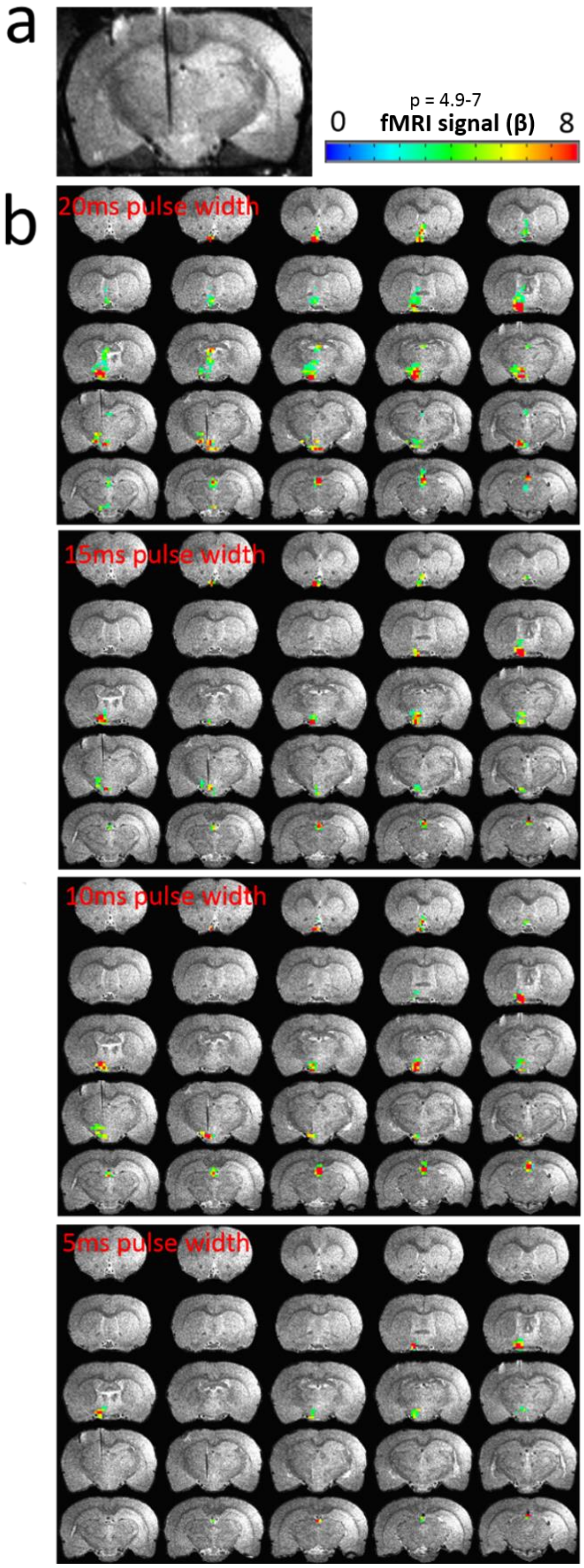

Supplementary Figure 5. Whole brain activity maps in response to 15s LH optogenetic stimulation at different pulse widths. **a** Anatomical image showing the position of the optical fiber for delivering light. **b** BOLD activation maps of a representative animal exhibited a pulse width-dependent pattern in response to 20ms, 15ms, 10ms and 5ms pulse widths (5 Hz, laser power of 12.6 mW, 15 s on 45 s off, 12 epochs). GLM-based t-statistics in AFNI is used.

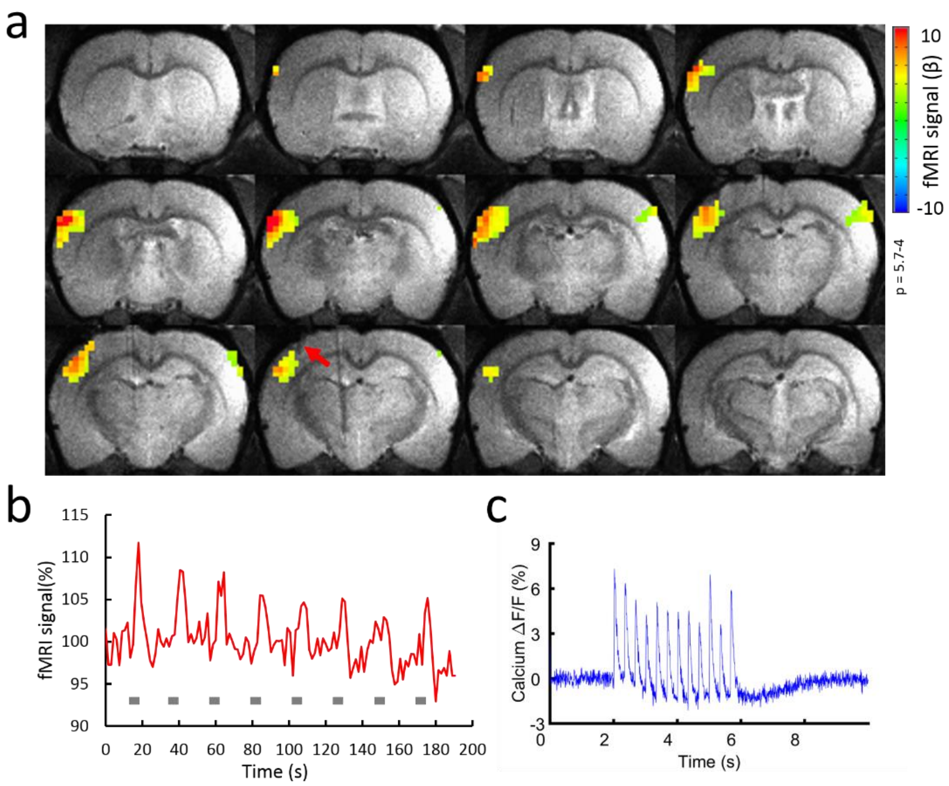

Supplementary Figure 6. Sensory-evoked neuronal Ca^{2+} recordings with simultaneous BOLD fMRI. **a** Representative color-coded BOLD-fMRI in response to a block design whisker-electrical stimulation. GLM-based t-statistics in AFNI is used. **b** The time course of evoked fMRI signal from BC-S1 ROI (see **a**) in the left hemisphere. **c** Average of simultaneously optical fiber (red arrow in **a**) recorded Ca^{2+} signals for one epoch (3 Hz, 4 s, 2 mA).

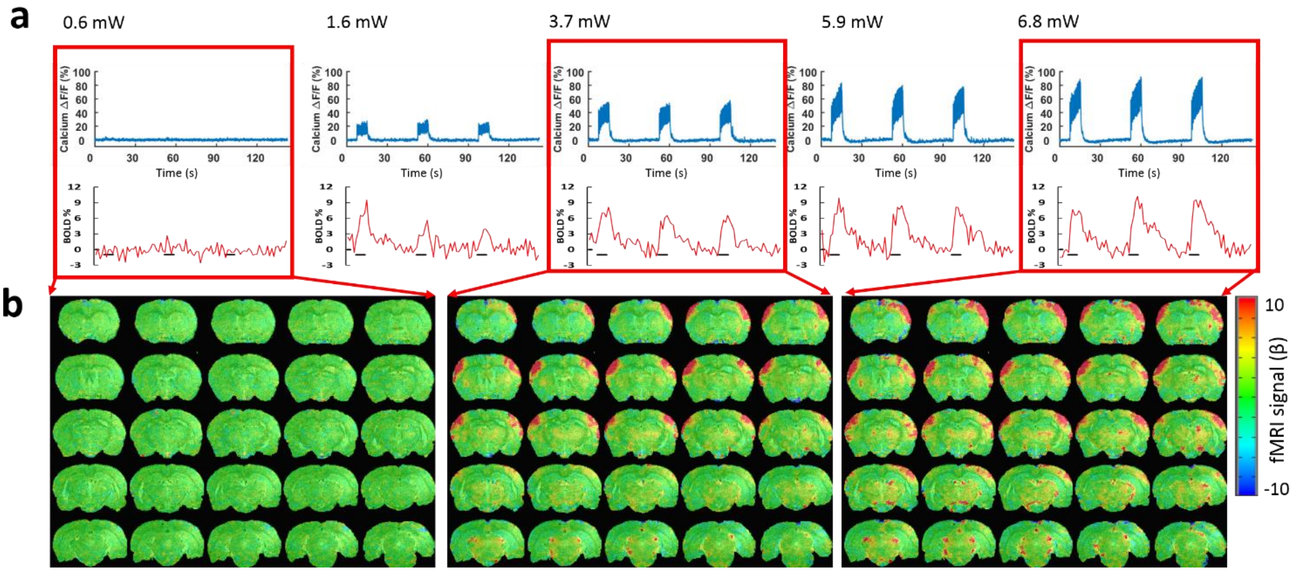

Supplementary Figure 7. Laser power dependent BOLD signals in S1 with simultaneous Ca^{2+} recordings (S1BF) upon light exposure in VPM. **a** Representative percentage changes of calcium signal (top) and BOLD responses (lower) for 3 epochs detected at 5 different laser powers. At 0.6 mW, hardly any fMRI and calcium signal was detected. BOLD and calcium signal increased proportionally with increased laser power. **b** Examples of whole brain activity maps at 0.6 mW, 3.7 mW and 6.8 mW.

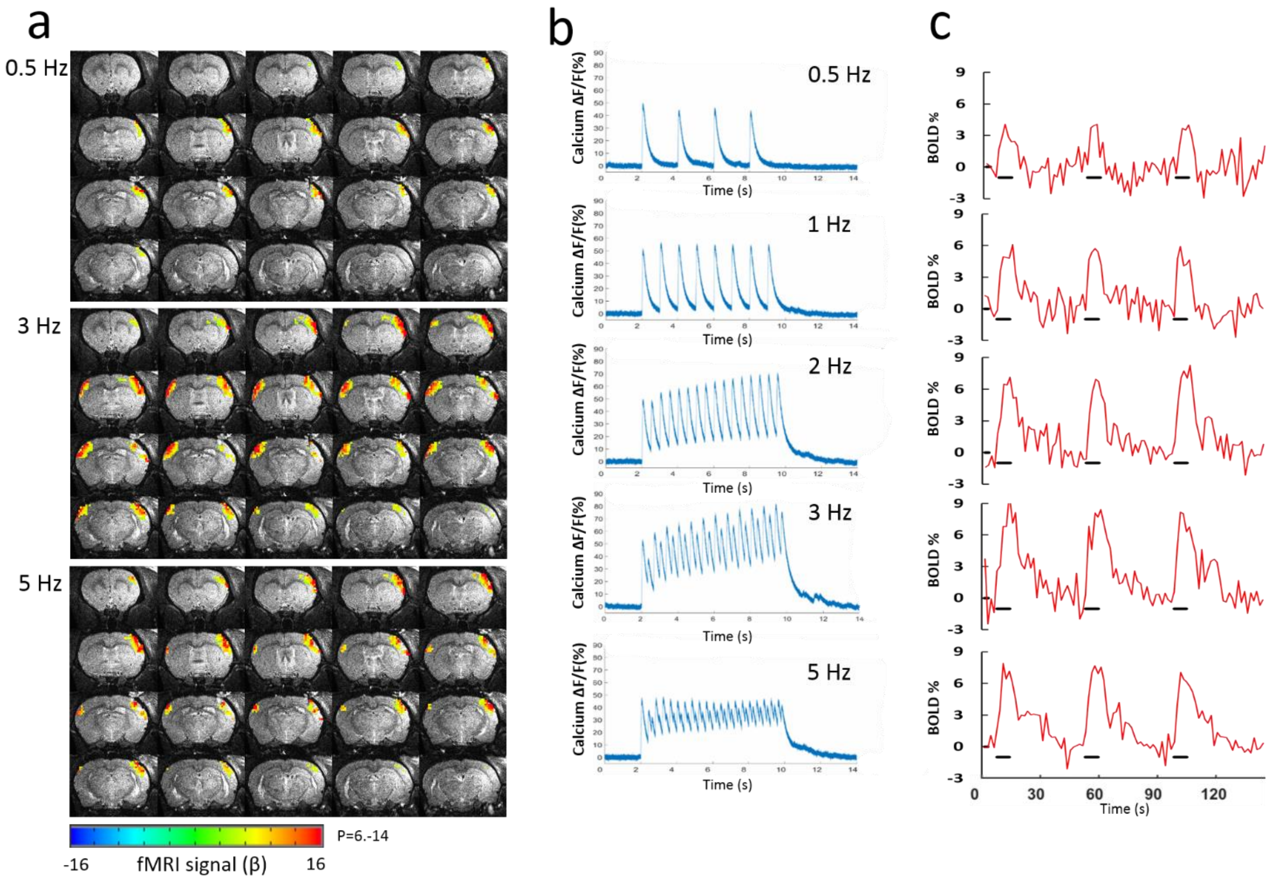

Supplementary Figure 8. Frequency dependent BOLD signals in S1 with simultaneous Ca^{2+} recordings (S1BF) upon light exposure in the Thalamus. **a** Examples of BOLD maps in response to 0.5 Hz, 3 Hz, 5 Hz. The strongest response was induced by 3 Hz stimulation, instead of 5 Hz. GLM-based t-statistics in AFNI is used. **b** Averaged calcium signal percentage change in one epoch. Evoked calcium spikes with almost full recovery to the baseline in 2 s per spike at 0.5 Hz. From 1 Hz to 3 Hz, the calcium signal was elevated through the 8 s stimulation period, while at 5 Hz, some of the spikes per pulse were even missed and the overall plateau amplitude was not further increased. **c** BOLD signal for 3 epochs upon stimulation (black line) was increased according to the increased frequency, but not at 5 Hz, which is consistent with the calcium signal observation.

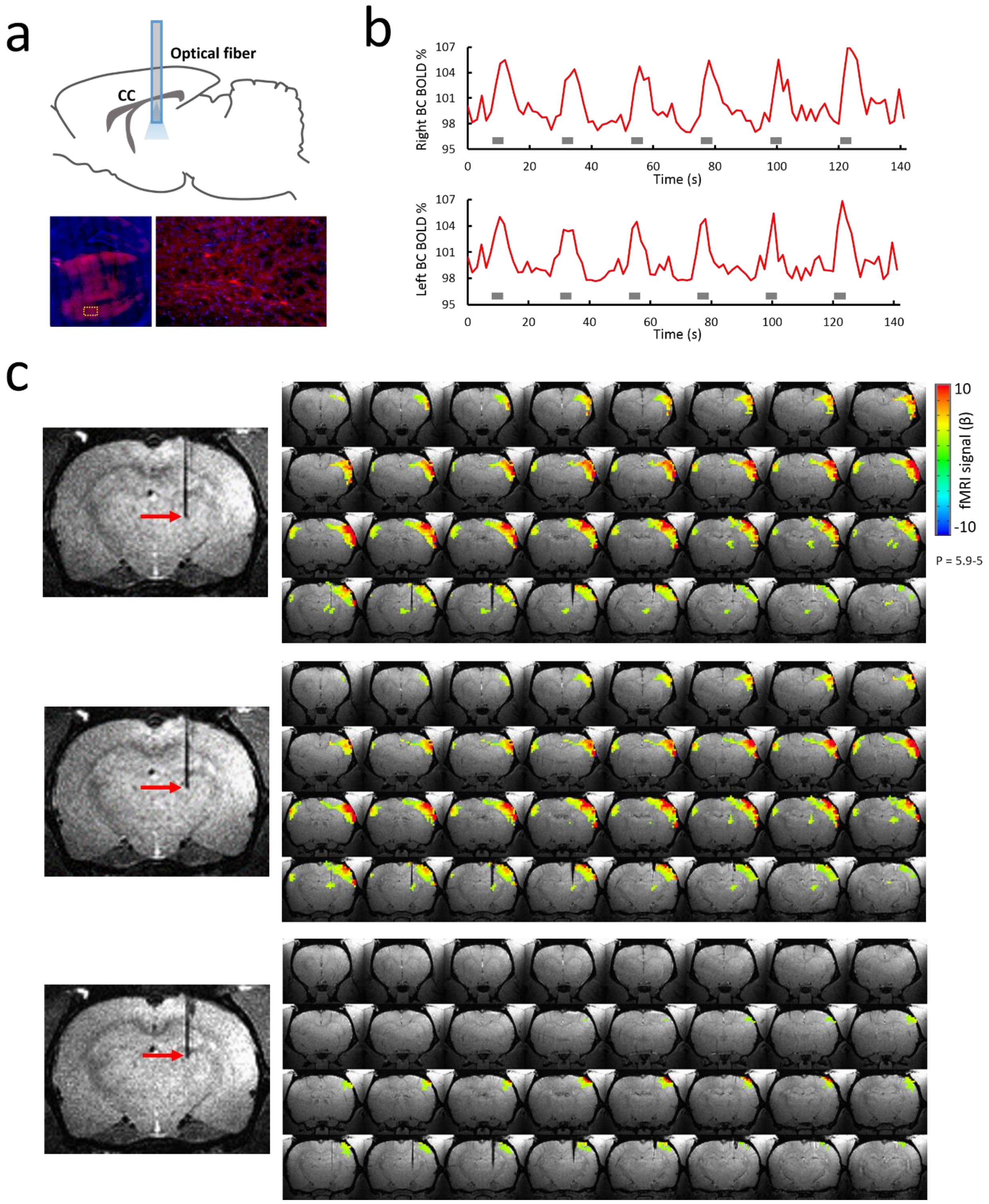

Supplementary Figure 9. Optogenetic excitation of thalamic cells drives local and Somatosensory cortical positive BOLD. **a** Top: Sketch showing the point of thalamic injection of AAV5.CAG.ChR2-mCherry and optical stimulation. Lower: histological image of ChR2-mCherry expression in the thalamus (left); higher magnification (right). Red, ChR2-mCherry; blue, 4′,6-Diamidin-2-phenylindol (DAPI). **b** Opto-fMRI haemodynamic response (averaged across activated voxels in Somatosensory cortical ROI, see **c**, whole brain top right) in both hemispheres during optical stimuli (5Hz, 4s on 18.5s off, 10 ms pulse width, laser power 5.5 mW). **c** BOLD activation at 3 different locations along the vertical insertion trajectory. GLM-based t-statistics in AFNI is used.

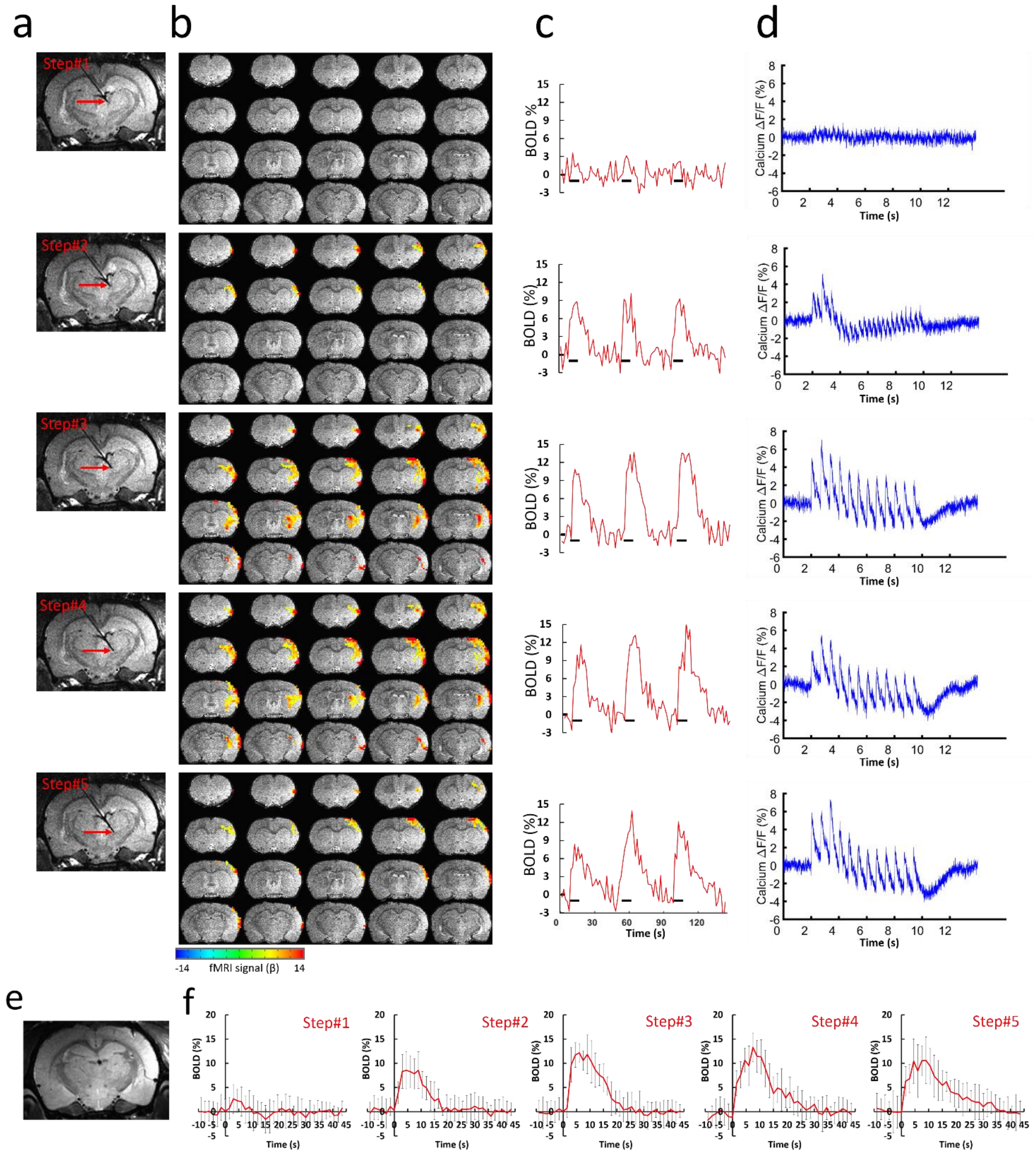

Supplementary Figure 10. Region-specific optogenetic activated neuronal Ca^{2+} recordings with simultaneous BOLD fMRI. **a** T2-weighted anatomical images illustrate five fiber locations. **b** Different BOLD fMRI in Somatosensory cortex evoked by optogenetic stimuli in different thalamic regions. **c** BOLD signals for 3 epochs (3 Hz, Laser power 4.2 mW, 8 s on 37 s off, 10 epochs) within ipsilateral Somatosensory cortex ROI (see b middle panel) corresponding to the different locations. **d** Simultaneously recorded evoked calcium signal through the 8 s stimulation period. **e** Anatomical RARE MR image illustrates the fiber tip location for calcium recording in Barrel cortex. **f** The average BOLD signals of ipsilateral hemisphere at different fiber tip locations. Error bars represent mean±SD.

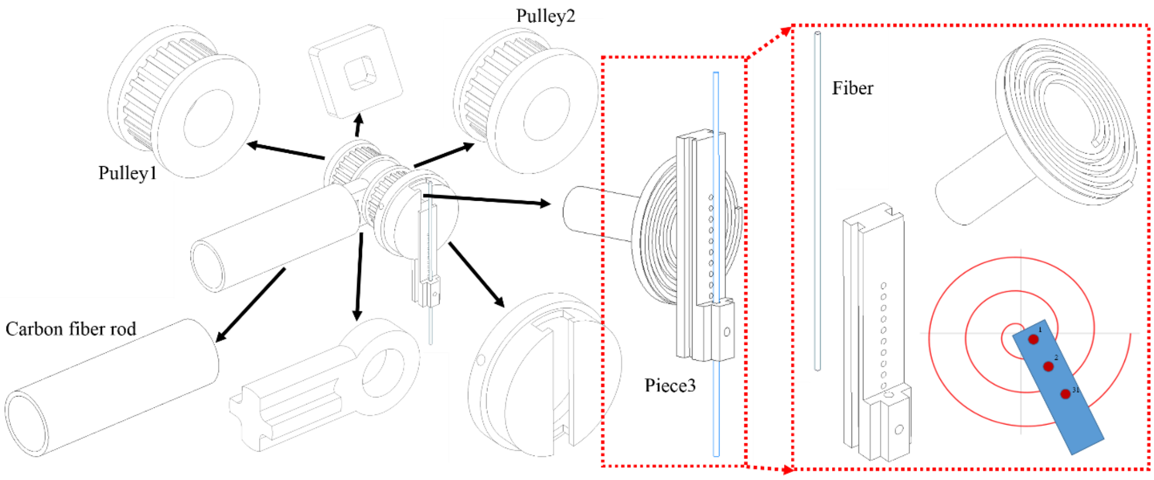

Supplementary Figure 11. Detailed design for the head part of the MgRA.

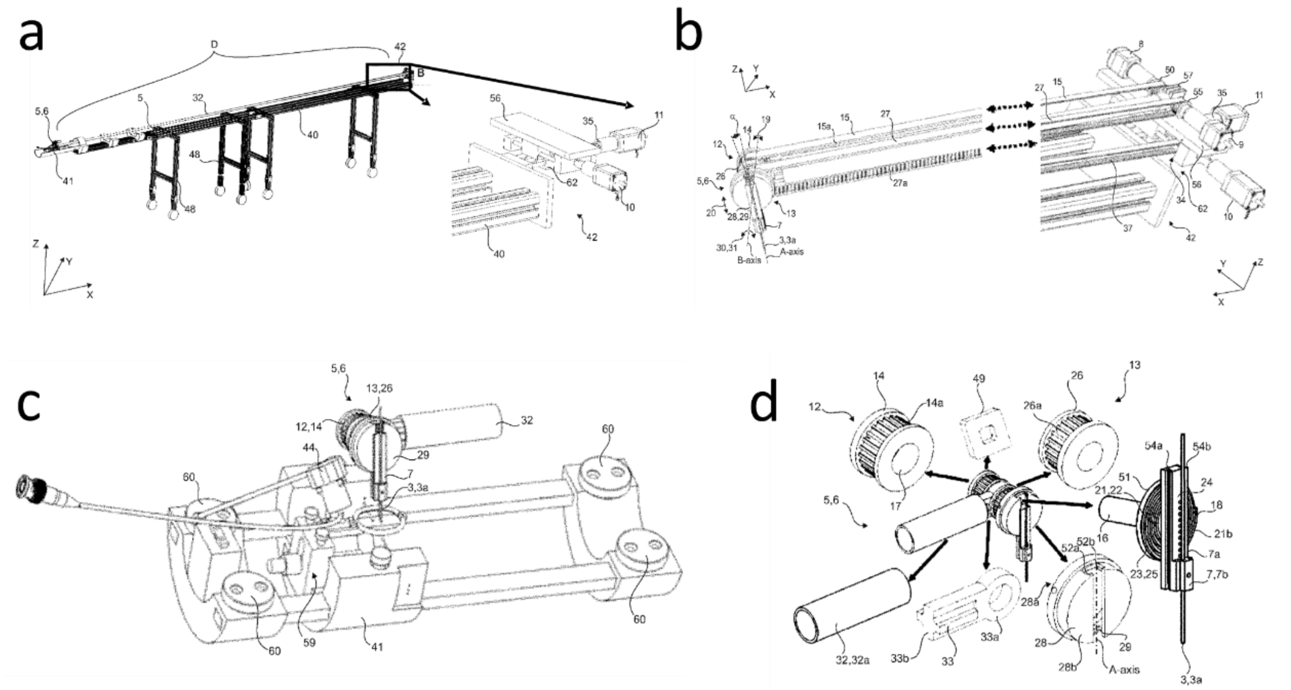

Supplementary Figure 12. Detailed design of the MgRA (figures from the approved European patent). **a** The schematic view of the whole MgRA mechanical design including the cross table to mount the stepper motors. **b** The coupling of the stepper motors (back part) to the matching toothed pulley in the head was achieved by a synchronous belt drive in a form-fit manner. **c** Custom-designed rat holder with a built-in MRI compatible camera, surface coil and head part of the MgRA. **d** The components of the head part of the MgRA. For more details see the approved European patent as following link: (https://patentscope.wipo.int/search/en/detail.jsf?docId=EP215319263&tab=PCTDESCRIPTION&maxRec=1000).

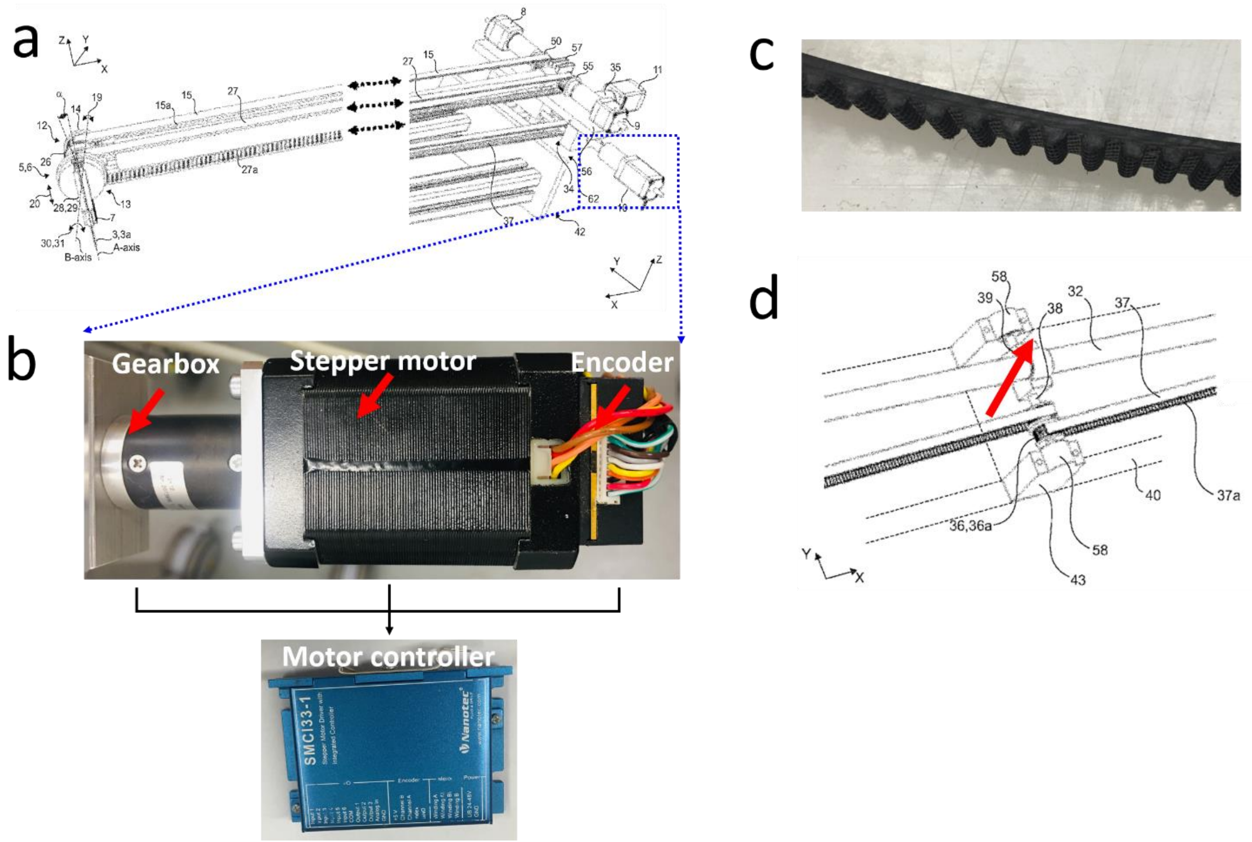

Supplementary Figure 13. Detailed design of the back part of the MgRA. **a** The coupling of the stepper motors (back part) to the matching toothed pulley in the head was achieved by a synchronous belt drive in a form-fit manner. **b** The encoder (NOE2-05-B14, Nanotec, Germany) is used with motor controller (SMCI33-1, Nanotec, Germany) so that the stepper motor (ST4118D1804-B, Nanotec, Germany) can be run in a close-loop mode. **c** Multi-groove belt (optibelt OMEGA 3M, Optibelt, Germany) used to fit into a matching toothed pulley. **d** Closed belts can be cascaded to transfer the motion (red arrow). All schematic figures shown here are from the approved MgRA European patent.
