# Supplementary material for: MRI-guided robotic arm drives optogenetic fMRI with concurrent Ca2+ recording
Source: Nat Commun. 2019 Jun 10;10:2536. doi: 10.1038/s41467-019-10450-3 (PMC6557837; doi:10.1038/s41467-019-10450-3)
Supplement: Supplementary file 3 — Description of Additional Supplementary Files [file 41467_2019_10450_MOESM3_ESM.pdf]

Description of Additional Supplementary Files

File name: Supplementary Movie 1

Description: 3D design of MgRA in "Inventor" (software package) environment.

File name: Supplementary Movie 2

Description: The mechanically controlled optical fiber inside the scanner videotaped by the built-in camera. An MRI-compatible camera was used to directly visualize the movement of the fiber (blue arrow, from dorsal to ventral), with the movement of the belt (red arrow) driven by a stepper motor mounted on the back part (Fig. 1c) of the robotic arm.

File name: Supplementary Movie 3

Description: Fiber insertion guided by MgRA with a step of 100 µm inside a perfused rat brain. Note the stability of the moving fiber.

File name: Supplementary Movie 4

Description: 50 µm stepsize movement for fiber optic insertion guided by MgRA with high stability.

File name: Supplementary Movie 5

Description: Fiber optic guidance outside of the rat brain. The built-in camera was used to guide the optical fiber to target the approximate location of the craniotomy. The video plays at 8x speed, the real speed of the fiber insertion is 20 µm per step.

File name: Supplementary Movie 6

Description: The location-dependent whole brain functional map during optical stimulation of the thalamus.

File name: Supplementary Movie 7

Description: The time-dependent Mn signal is highly confined close to the injection area with limited diffusion.
