# Supplementary material for: MRI-guided robotic arm drives optogenetic fMRI with concurrent Ca2+ recording
Source: Nat Commun. 2019 Jun 10;10:2536. doi: 10.1038/s41467-019-10450-3 (PMC6557837; doi:10.1038/s41467-019-10450-3)
Supplement: Supplementary file 11 — Reporting Summary [file 41467_2019_10450_MOESM11_ESM.pdf]

natureresearch

Corresponding author(s): Xin Yu
Last updated by author(s): Apr 8, 2019

Reporting Summary

Nature Research wishes to improve the reproducibility of the work that we publish. This form provides structure for consistency and transparency in reporting. For further information on Nature Research policies, see Authors & Referees and the Editorial Policy Checklist.

Statistics

For all statistical analyses, confirm that the following items are present in the figure legend, table legend, main text, or Methods section.

n/a	Confirmed	
☐	☒	The exact sample size (*n*) for each experimental group/condition, given as a discrete number and unit of measurement
☐	☒	A statement on whether measurements were taken from distinct samples or whether the same sample was measured repeatedly
☐	☒	The statistical test(s) used AND whether they are one- or two-sided<br>*Only common tests should be described solely by name; describe more complex techniques in the Methods section.*
☒	☐	A description of all covariates tested
☒	☐	A description of any assumptions or corrections, such as tests of normality and adjustment for multiple comparisons
☐	☒	A full description of the statistical parameters including central tendency (e.g. means) or other basic estimates (e.g. regression coefficient) AND variation (e.g. standard deviation) or associated estimates of uncertainty (e.g. confidence intervals)
☒	☐	For null hypothesis testing, the test statistic (e.g. *F*, *t*, *r*) with confidence intervals, effect sizes, degrees of freedom and *P* value noted<br>*Give P values as exact values whenever suitable.*
☒	☐	For Bayesian analysis, information on the choice of priors and Markov chain Monte Carlo settings
☒	☐	For hierarchical and complex designs, identification of the appropriate level for tests and full reporting of outcomes
☒	☐	Estimates of effect sizes (e.g. Cohen's *d*, Pearson's *r*), indicating how they were calculated

Our web collection on statistics for biologists contains articles on many of the points above.

Software and code

Policy information about availability of computer code

Data collection	For MRI data, ParaVision®5 - Bruker, Germany<br>For calcium, AcqKnowledge Acquisition & Analysis Software, BIOPAC system (MP150 System, BIOPAC Systems, USA).
Data analysis	P18<br>AFNI(Analysis of Functional NeuroImages), NIH<br>MATLABR2016a

For manuscripts utilizing custom algorithms or software that are central to the research but not yet described in published literature, software must be made available to editors/reviewers. We strongly encourage code deposition in a community repository (e.g. GitHub). See the Nature Research guidelines for submitting code & software for further information.

Data

Policy information about availability of data

All manuscripts must include a data availability statement. This statement should provide the following information, where applicable:

- Accession codes, unique identifiers, or web links for publicly available datasets
- A list of figures that have associated raw data
- A description of any restrictions on data availability

The raw data can be provided upon email request to the corresponding author and excel files are included for each quantitative plot included in the main figures. For the design of the robotic arm, detailed infor-mation can be directly downloaded through the official link of World Intellectual Property Organization (WIPO):https://patentscope.wipo.int/search/en/detail.jsf?docId=EP215319263&tab=NATIONALBIBLIO&maxRec=1000.

Field-specific reporting

Please select the one below that is the best fit for your research. If you are not sure, read the appropriate sections before making your selection.

☒ Life sciences ☐ Behavioural & social sciences ☐ Ecological, evolutionary & environmental sciences

For a reference copy of the document with all sections, see nature.com/documents/nr-reporting-summary-flat.pdf

Life sciences study design

All studies must disclose on these points even when the disclosure is negative.

Sample size	A total of 21 male Sprague–Dawley rats were used in this study. P19
Data exclusions	For rats died during the middle of fMRI experiments, the data were not included for analysis.
Replication	Yes. See Figure 3.
Randomization	N/A
Blinding	N/A

Reporting for specific materials, systems and methods

We require information from authors about some types of materials, experimental systems and methods used in many studies. Here, indicate whether each material, system or method listed is relevant to your study. If you are not sure if a list item applies to your research, read the appropriate section before selecting a response.

Materials & experimental systems

n/a	Involved in the study	
☒	☐	Antibodies
☒	☐	Eukaryotic cell lines
☒	☐	Palaeontology
☐	☒	Animals and other organisms
☒	☐	Human research participants
☒	☐	Clinical data

Methods

n/a	Involved in the study	
☒	☐	ChIP-seq
☒	☐	Flow cytometry
☐	☒	MRI-based neuroimaging

Animals and other organisms

Policy information about studies involving animals; ARRIVE guidelines recommended for reporting animal research

Laboratory animals	A total of 21 male Sprague–Dawley rats were used in this study. P14
Wild animals	N/A
Field-collected samples	N/A
Ethics oversight	The study was performed in accordance with the German Animal Welfare Act (TierSchG) and Animal Welfare Laboratory Animal Ordinance (TierSchVersV). This is in full compliance with the guidelines of the EU Directive on the protection of animals used for scientific purposes (2010/63/EU). The study was reviewed by the ethics commission (§15 TierSchG) and approved by the state authority (Regierungspräsid-ium, Tübingen, Baden-Württemberg, Germany). P14

Note that full information on the approval of the study protocol must also be provided in the manuscript.

Magnetic resonance imaging

Experimental design

Design type	Task, Block design
Design specifications	P18
Behavioral performance measures	N/A

Acquisition

Imaging type(s)	P17-18
Field strength	P3, 14.1 T
Sequence & imaging parameters	P17-18
Area of acquisition	P6, a whole brain scan was used.

Diffusion MRI ☐ Used ☒ Not used

Preprocessing

Preprocessing software	AFNI, P19
Normalization	Included in the standard AFNI code.
Normalization template	Included in the standard AFNI code.
Noise and artifact removal	Included in the standard AFNI code.
Volume censoring	N/A

Statistical modeling & inference

Model type and settings	Included in the standard AFNI code.
Effect(s) tested	N/A

Specify type of analysis: ☐ Whole brain ☐ ROI-based ☒ Both

Anatomical location(s): Lateral hypothalamus and barrel cortex. Based on brain atlas (Paxinos&Watson), sixth edition.

Statistic type for inference (See Eklund et al. 2016)	Cluster-wise (>20 voxels). Detailed p-values are included.
Correction	FDR based correction.

Models & analysis

n/a	Involved in the study
☒	☐ Functional and/or effective connectivity
☒	☐ Graph analysis
☒	☐ Multivariate modeling or predictive analysis
